# Preliminary Evidence for the Emergence of a Health Care Online Community of Practice: Using a Netnographic Framework for Twitter Hashtag Analytics

**DOI:** 10.2196/jmir.7072

**Published:** 2017-07-14

**Authors:** Damian Roland, Jesse Spurr, Daniel Cabrera

**Affiliations:** ^1^ SAPPHIRE Group Health Sciences Leicester University Leicester United Kingdom; ^2^ Emergency Department Redcliffe Hospital Brisbane Australia; ^3^ Department of Emergency Medicine Mayo Clinic Rochester, MN United States

**Keywords:** social media, network, community networks, community of practice, #FOAMed, Twitter

## Abstract

**Background:**

Online communities of practice (oCoPs) may emerge from interactions on social media. These communities offer an open digital space and flat role hierarchy for information sharing and provide a strong group identity, rapid flow of information, content curation, and knowledge translation. To date, there is only a small body of evidence in medicine or health care to verify the existence of an oCoP.

**Objective:**

We aimed to examine the emergence of an oCoP through the study of social media interactions of the free open access medical education (FOAM) movement.

**Methods:**

We examined social media activity in Twitter by analyzing the network centrality metrics of tweets with the #FOAMed hashtag and compared them with previously validated criteria of a community of practice (CoP).

**Results:**

The centrality analytics of the FOAM community showed concordance with aspects of a general CoP (in terms of community, domain, and practice), as well as some specific traits of a health care community, including social control, common purpose, flat hierarchy, and network-based and concrete achievement.

**Conclusions:**

This study demonstrated preliminary evidence of an oCoP focused on education and based on social media interactions. Further examination of the topology of the network is needed to definitely prove the existence of an oCoP. Given that these communities result in significant knowledge translation and practice change, further research in this area appears warranted.

## Introduction

Creation, dissemination, and management of knowledge are cornerstones of safe and effective health care, but achieving these goals in functional and successful large care systems remains enormously challenging. Within this construct of health systems [1], the concept of a network is vital for improving patient care by fostering collaboration, stimulating engagement and knowledge acquisition and management, and promoting learning [2]. Recently, social media-based platforms have been proffered as educational tools [3,4], based on the critical assumption that activity in online networks can lead to the emergence of personal learning networks [5] and communities of practice (CoPs) [6].

CoPs are an anthropological concept, defined as a group of people who organize around a specific component of knowledge (eg, a profession or particular task). They create, organize, and share information and not only develop specific domains but also foster development of individual members [6]. These communities are organized around a domain, community, and practice, where the domain is a realm of knowledge that the members of the group have interest in and value as important; the community is the coalescence of interactions and negotiations between members around the domain; and practice is the results of the cooperation leading to the creation of tangible resources affecting their practice of the knowledge. The presumed natural evolution of organically organized online communities [7] is the description and identification of an online CoP (oCoP) [8]. This construct offers an enormous potential for the creation, dissemination, and management of knowledge and people using a relatively minimal amount of resources and appearing as an ideal framework for information-oriented teams. These have arisen from the Internet in general and particularly in social media platforms [9] because of their ability to efficiently connect and engage groups, provide peer-based education [10], and manage meaningful knowledge [8].

The concept of CoP has been thoroughly described in health care [11], and the critical components of an appropriate network were refined and expanded by Aveling et al in 2012 [12]. A health care CoP is defined by 8 core characteristics (described below) that are themselves derived from Wenger’s [6] pivotal components of community, domain, and practice.

Although CoPs are not a new phenomenon, social media platforms have enabled them to form rapidly and across international boundaries, with nonlinearity and on a large scale [13]. These new oCoPs create discrete and quantifiable data flow among users (nodes) of the network. Knowledge and relationships develop by these interactions (links), with functional communities arising as information transfer increases. These communities of online learning and practice offer an open digital space for information sharing, with a flat role hierarchy, strong group identity, high engagement, and rapid flow of information, content curation, and knowledge translation. In many ways, their topology represents the structure of scale-free networks [14,15]. The use of social media platforms as an educational medium is dependent on these oCoPs providing reliable and manageable information for their participants [16].

Despite the recognition of several possible candidates for CoPs and their derivatives in online platforms [8], particularly in the health care sector [11] and for peer-to-peer patient interactions [17], medical learning and practice oCoPs have been rarely described in the medical literature to date. An international movement that began within the past 5 years now aims to collaborate to create, curate, and disseminate medical knowledge with the intention of changing patterns of care; an explicit aim is to reduce the knowledge translation gap [2,15]. This movement describes the concept as “free open access medical education” (FOAM) [18,19] and organizes around the Twitter hashtag #FOAMed; the group name is synonymous with and represents its core philosophy.

The strength of oCoPs can be established if these communities are identified and their relationships described; social media education could then be validated as a viable paradigm for knowledge translation [16] that affects health care practice. The aim of this study was to show preliminary evidence of the emergence of a health care oCoP focused on knowledge translation and organized as a network architecture around the Twitter hashtag #FOAMed. By using data extracted from our #FOAMed hashtag analysis, we aimed to demonstrate that the FOAM community fulfills the core characteristics of a CoP, as described by Wenger [6] and expanded by Aveling et al [12], and is a community of individuals with different roles who belong to different organizations and places, who organize themselves around a concrete domain and specific goal, and who function to support and promote each other’s development to achieve change.

We hypothesized that the FOAM community, defined by the explicit interactions that include the #FOAMed hashtag, constitutes an oCoP. To confirm this, we believed it should be possible to recognize the emergence of the community, domain, and practice of the network, while describing information flowing between nodes and the semantic relationships between the members. Describing the structure of the network, as well as the influences of nodes outside the network (eg, spambots), requires a robust intrinsic familiarity with the community; therefore, we approached the analysis using a netnographic methodology [20,21] based on our personal participation in the FOAM movement.

## Methods

The challenge of analyzing billions of interactions in social media [22,23] is a relatively recent area of activity that requires a high level of expertise and computational power. We created a Symplur Signals database (Symplur LLC, Upland, CA, USA) around the Twitter hashtag #FOAMed and interrogated it from March 1, 2013, through August 31, 2015; this dataset contained almost all data available for the hashtag because the accepted inception time for #FOAMed was March 2013 and the activity occurred explicitly around the hashtag [18]. The computational tools implemented by Symplur Signals have been validated previously for the analysis of Tweet chats [24]. There is a large amount of FOAM community activity, such as blogs, podcasts, and conference proceedings, that is not directly related to the #FOAMed hashtag or Twitter; however, these products are typically references in community activity and it is highly unlikely that such activity outside of Twitter would nullify the existence of the oCoP.

**Table 1:** Aveling’s core components of clinical communities of practice and their relationship with Wenger’s classic definition.

Composite postulate notation	Aveling core components of clinical communities^a^	#FOAMed proof	Wenger definition^b^
A1C	Consists of interdependent groups and individuals	The #FOAMed hashtag connects individuals who demonstrate interactions with each other. Over time, influencers increase in number and become divergent rather than convergent.	Community
A2C	Consists of members who may cross clinical and organizational boundaries	#FOAMed hashtag is used by a variety of individuals and organizations. Over time, geographic area of use increases.	Community
A3D	Consists of members united by a common purpose of bridging the gap between best scientific evidence and current clinical practice	Content of #FOAMed remains around health care-related themes, centered on creation and access to content.	Domain
A4CDP	Consists of members who come together not only to learn or share knowledge but to achieve those aims	Discussion around #FOAMed results in positive attributions regarding content.	Community, domain, practice
A5DCP	Exploits the networks’ inherent potential for effective and low-cost knowledge generation and diffusion	#FOAMed generates subnetworks around individuals or content nodes (such as specific websites).	Domain, community, practice
A6C	Operates through both vertical and lateral structures	The network expands through increasing individuals who influence others across increasingly wide geographic areas.	Community
A7CP	Deploys peer influence and uses primarily informal, social control mechanisms to achieve change	Key nodes exert influence, but this changes through time.	Community, practice
A8P	Harnesses the power of the community and its collective wisdom when seeking solutions to problems; includes contextual factors and local solutions	Interactions (measured through mentions) expand rather than contract over time.	Practice

^a^Adapted from Aveling et al [12]. Used with permission.

^b^Data from Wenger [6].

To reduce potential bias in the analysis, we submitted a protocol to the Symplur Signals team defining the a priori analytic strategy. Within this protocol, we proposed several proofs to demonstrate concordance with the Aveling-Wenger postulate for a CoP. Table 1 lists these proofs, matched to the 8 distinct core characteristics. For example, evidence of interdependent groups and individuals (A1C) would be demonstrated by showing that the #FOAMed hashtag was used by individuals who had interactions with each other and that, over time, influencers increased in number, becoming divergent rather than convergent. To do this, we would therefore need to describe #FOAMed hashtag use quantitatively. Conceptually, we aimed to analyze #FOAMed hashtag activity as a network. The hashtag [25] constitutes the core scaffold in which the identified social activity occurs. The network consists of Twitter users (nodes), and interactions (links) with specific metrics of interactions such as retweets, favorites, and engagements, which constitute the connections and distribution by which networks are typically constructed [26,27].

Given the social nature of the Twitter platform, we used the social network analysis component of the netnographic framework for analysis, as described by Kozinets [20]; this method is particularly effective at identifying external influences that are not meaningful to the community (eg, spambots, large commercial interests), as well identifying digital artefacts that are created by mistake or are otherwise inappropriate (eg, links to unrelated events associated with the hashtag but used by a completely different community). From a netnographic perspective, it is difficult to appreciate the metrics of centrality without a personal understanding of the symbolism, meaning, and consumer patterns of the groups [28]; however, as members of the community of study, we had an immersive and descriptive understanding of the FOAM movement. Our intimate understanding allowed us to determine (via a consensus view of the authors) the relevance of the top 100 shared links identified in the ethnographic analysis of the FOAM community We generally excluded broken links, links to landing pages of websites or home pages, and any other links that did not point to a webpage with a publicly visible forum for comments and feedback or had no comments posted. This study was deemed exempt from ethics board review by the English National Health Service Health Research Authority.

## Results

**Figure 1:**
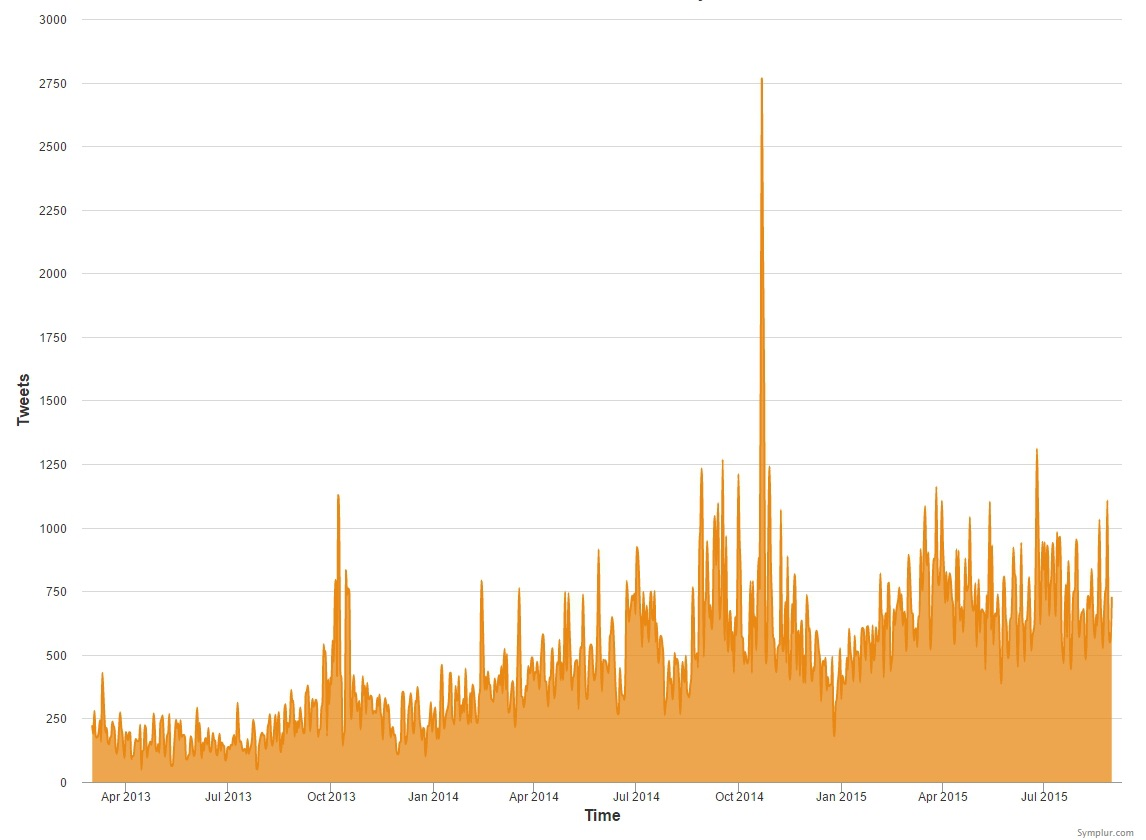
Tweet activity (number of tweets per day) using the #FOAMed hashtag from March 1, 2013, through August 31, 2015.

**Figure 2:**
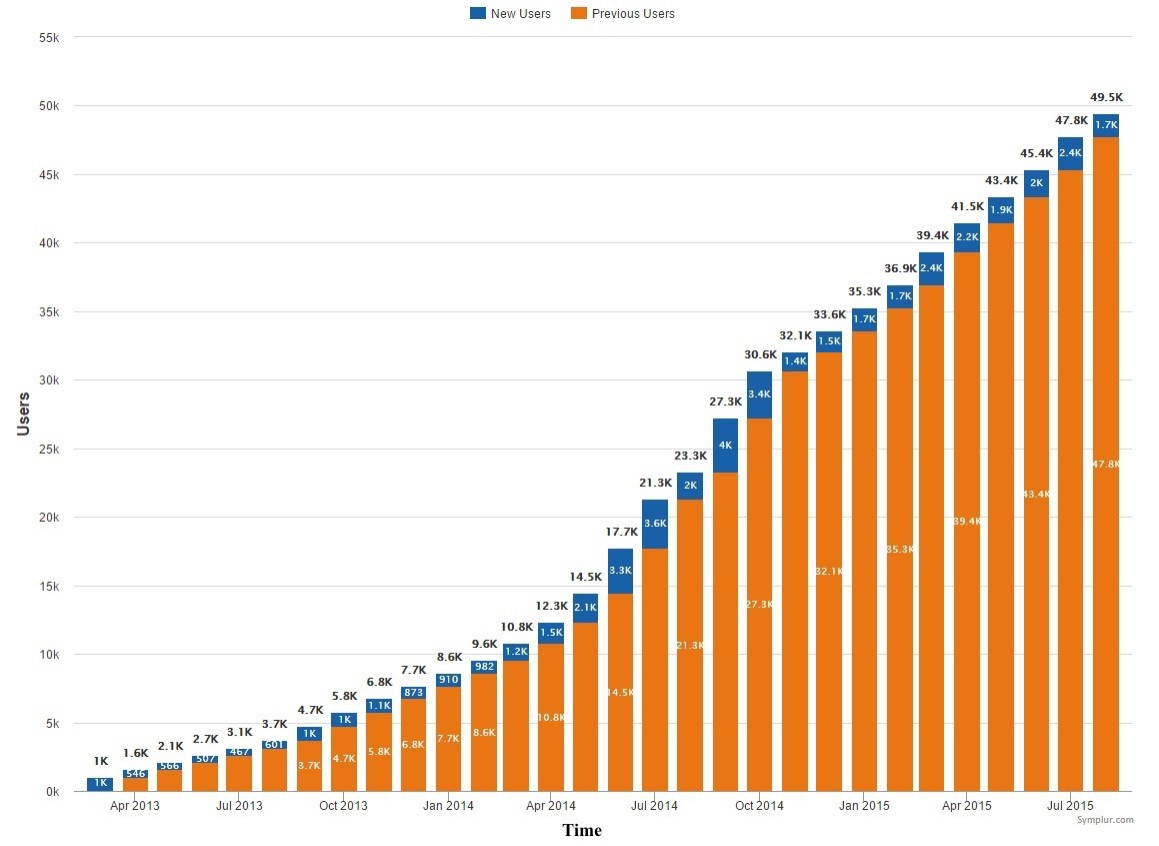
Cumulative number of users participating in the #FOAMed community.

During the study period, we identified 49,459 active users who issued 429,606 tweets and created 1,258,692,900 impressions (ie, the number of times a user is served a Tweet in their timeline or search results); this translated to more than 8 tweets and 25,000 impressions per user involved in the conversation (Table 2). The user and hashtag activity, expressed as number of daily tweets, increased during the study period (from ~250 to ~700 tweets/day) (Figure 1). User participation increased substantially during the same time frame, rising from around 1000 users to more than 45,000; an additional 1000 users per month have participated since March 2014 (Figure 2). Multimedia Appendix 1 shows the top influencers. In terms of engagement, 27,635 users (55.71%) participated with 1 tweet (representing 6.43% of all tweets), while 2603 users (5.25%) tweeted more than 20 times and were responsible for 72.45% of all tweets (Table 3).

**Figure 3:**
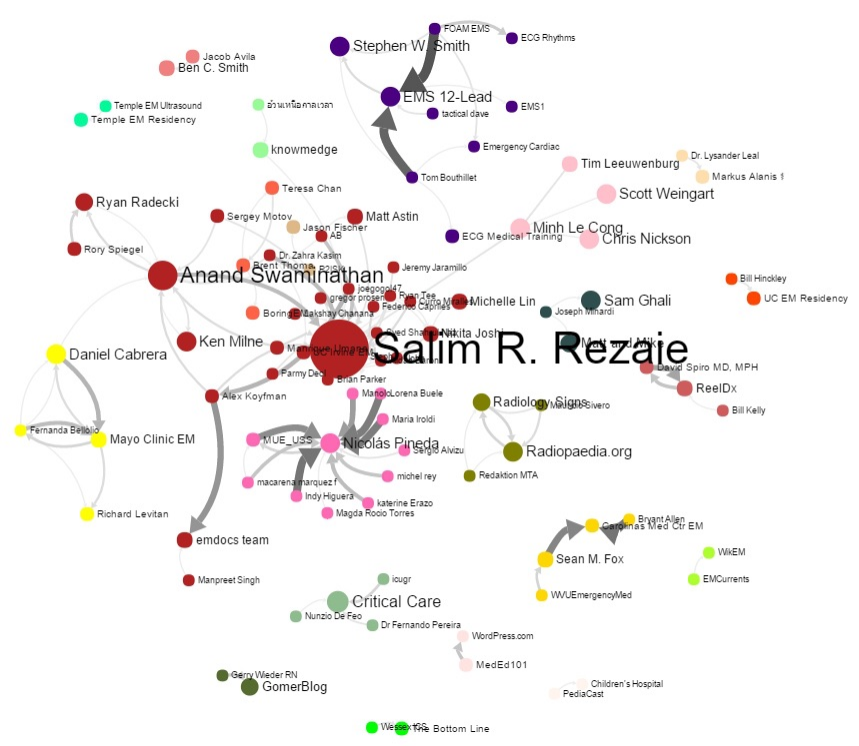
Partial graphic depiction of the centrality metrics of the top 100 #FOAMed users based on weight (defined by mentions, hub, and authority quotients).

**Figure 4:**
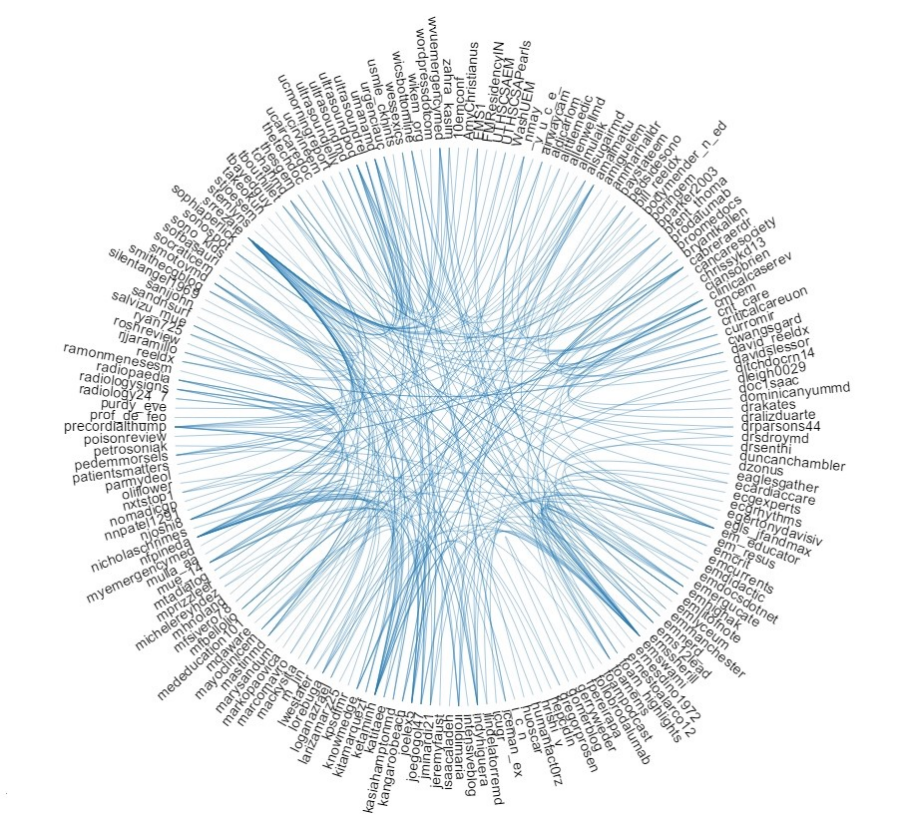
Conversation identifier depicting frequency and strength of interaction among top members of the #FOAMed community.

From a network distribution perspective, the top 200 users created 148,185 tweets, representing 34.49% of all tweets of the community. The Symplur Signals analytics algorithm created a centrality map by considering nodes (users) and their interactions (links) to assign weight and used the metrics of mentions, authority, and hub to assign the weight of centrality [29,30]. The 100 users with the highest weights also tended to have high authority and edge scores (Multimedia Appendix 2), and from a netnographic perspective, they were recognizable as community leaders. Figure 3 outlines their status in relation to the number of interactions with other participants in a graphical representation, where the size of the node represents its network centrality (relative influence) and the links depict the strength and directions of the interactions between members (nodes) of the network. Also, Figure 4 depicts a perspective of the communication between nodes on a conversation matrix showing the frequency and strength of interactions among the most active community members.

**Table 2:** Metrics of the Twitter hashtag #FOAMed^a^.

Metric	Total	No. per month	No. per week	No. per day	No. per hour
Tweets	429,606	14,132	3298	471	20
Users who tweeted	49,459	1627	380	54	2
Tweets per user	8.69	0.29	0.07	9.52×10^−3^	3.97×10^−4^
Impressions	1.26×10^9^	4.14×10^7^	9.66×10^6^	1.38×10^6^	5.75×10^4^
Impressions per user	25,447	837	195	28	1

^a^Hashtag activity was monitored from March 1, 2013, through August 31, 2015.

**Table 3:** Twitter engagement metrics for the #FOAMed hashtag^a^.

User activity by no. of tweets	Total users, n (%)	Proportion of all tweets, %
1	27,635 (55.71)	6.43
2	7078 (14.27)	3.30
3	3703 (7.46)	2.59
4	2103 (4.24)	1.96
5	1366 (2.75)	1.59
6	1011 (2.04)	1.41
7	698 (1.41)	1.14
8	602 (1.21)	1.12
9	454 (0.92)	0.95
10	382 (0.77)	0.89
11	326 (0.66)	0.83
12	269 (0.54)	0.75
13	229 (0.46)	0.69
14	182 (0.37)	0.59
15	164 (0.33)	0.57
16	164 (0.33)	0.61
17	145 (0.29)	0.57
18	122 (0.25)	0.51
19	118 (0.24)	0.52
20	110 (0.22)	0.51
>20	2603 (5.25)	72.45

^a^Total tweets, 429,606; total number of users who tweeted, 49,459.

**Table 4:** Geographic location of #FOAMed community members (n=9502)^a^.

Country	n (%)
United States	4137 (43.5)
United Kingdom	1926 (20.3)
Australia	820 (8.6)
Canada	797 (8.4)
Spain	434 (4.6)
Russia	384 (4.0)
Brazil	270 (2.8)
Mexico	270 (2.8)
India	264 (2.8)
Saudi Arabia	200 (2.1)

^a^Not all users self-identified their locations.

In terms of geographic distribution, the community members generally lived in anglophone Western countries, although some of them were located in Latin American and Asian countries (Table 4). However, the analytic tool was unable to consider the geographic location of more than 50% of users because they did not provide self-identified geolocation information.

Multimedia Appendix 3 details the top 150 conversations (defined by Symplur in terms of level of engagement) generated in the community during the study period. The users involved were those with the highest network weight and hub index. The conversations showed a relatively low level of branching, with most having 2 or 3 levels of interactions (replies). An automatic sentiment analysis [31] showed a 0.367 positive sentiment embedded in the tweets. In terms of knowledge sharing, an analysis of the top 100 links (Multimedia Appendix 4) shared by the group shows a mix of self-created content (blogs), referral to social platforms (eg, YouTube, Vine), and referral to traditional media (eg, website for the *New England Journal of Medicine*). We analyzed a total of 26 blogs (Multimedia Appendix 5) to explore the contextual meaning of the #FOAMed community. Ethnographic examination showed forums of discussion by way of blog comments, with commenters also being members of the #FOAMed Twitter community.

## Discussion

### Principal Findings

Analysis of the data revealed evidence of a community organized around a scale-free topology network built on the hashtag #FOAMed, with an increasing number of users (nodes) and connections (links) manifested as impressions. The community is organized around members with a high degree of influence (centrality) that functions as a major hub for communication and knowledge management. In terms of content, knowledge appears to have a similar architecture, with relatively small pieces (blog posts or videos) concentrating most of the interactions and functioning also as hubs with high centrality [32,33].

The outcomes of the #FOAMed hashtag database analysis can be matched against the composite Aveling-Wenger [6,12] postulate for a health care CoP (Table 1). Thus, there is preliminary evidence suggesting the #FOAMed community possesses many of the characteristics of an oCoP and may contribute to recent literature describing learning and practice oCoPs [34].

In terms of the formation of interdependent groups and individuals (A1C) and the crossing of clinical and organizational boundaries (A2C), the data support the existence of a diverse group of people, organized around content and users hubs (Tables 1 and 2); the community includes health care providers in different roles (physicians, nurses, paramedics, etc), as well as health care and educational organizations; and there is robust interaction between users (nodes) of different background, trainings, and specialties. At the time of analysis, community members were mostly located in Western anglophone countries, with fewer members in Asia and Latin America (Multimedia Appendix 4). However, while it is possible to describe the volume of the dependencies, it is more challenging to demonstrate their meaning. It must be acknowledged that, due to the size of the community, it is difficult to completely verify the relationships and interactions that prove the interdependencies suggested by the Aveling-Wenger postulate. The analysis of an oCoP focuses on the members of the community and their interactions around pieces of the domain in order to construct a practice and is essentially different from a network analysis. This approach, although very relevant, would not have been appropriate for this work, given the lack of focus on content. Clearly, community members are engaged in the dissemination and sharing of medical knowledge, as demonstrated by the type and frequency of link sharing and the efforts of the community to increase the reach of the information through retweets (Multimedia Appendix 4). It is key to describe that all of the content analyzed was quite concentrated on medical knowledge, there were no links to nonbiomedical content, and the discussions were in a high degree quite focused on dissemination and critique; in other words, there was very minimal noise in the discussions among the members of the community (Multimedia Appendix 3). These actions embody the core of the common purpose postulate (A3D) and the exploitation of inherent networks (A5DCP). However, the nature of those networks is not clear and diffusion of knowledge cannot be encapsulated using this whole-system review approach, which is a limitation of a digital-based analysis of knowledge translation.

The most powerful characteristic of the #FOAMed network [27] is the robustness and richness of the interactions between the users (nodes) through strong relations (links); this activity resulted in more than a billion relations (retweets or links) [30], with a clear delineation of network synergy (Figures 3 and 4; Table 4), which support achievement as well as learning and sharing knowledge (A4CDP) and vertical and lateral structure operation (A6C). The centrality maps show a classic scale-free topology, with clear hubs for content and users arranged in a mesh topology, illustrating a vertical and horizontal arrangement in the Aveling construct.

The measures of weight based on hub and authority showed evidence of peer influence and online social mechanisms that influence peers and community members and create subcommunities (Figures 3 and 4); this suggests social control mechanisms (postulate A7CP) and, combined with the activity related to link and knowledge, suggests evidence of harnessing the power and collective wisdom of the community (A8P) as shown in the netnographic analysis in Multimedia Appendix 3. This describes the top 150 conversation threads and demonstrates the branching structure, each with up to 15 different users and as many as 8 branches. A very small number of conversations with very few participants would have challenged the postulate that users all have a contribution to make. We acknowledge our analysis of the #FOAMed community appears to show centralization around a few clusters of influence and not a completely distributed architecture. This appears to be related to coalescence of users around sources of knowledge and does imply that there are core users of the network as well as those at the periphery.

The primary aim of this research was to show preliminary evidence of the emergence of an oCoP from the FOAM movement represented by Twitter interactions that included the #FOAMed hashtag. The concept of CoP was introduced by Wenger [6] and describes the appearance of networks of people who interact explicitly, create and negotiate knowledge, and are able to translate this knowledge into a praxis. Our analysis indicates the possible emergence of a community, as evidenced by the large number of users, with a clear level of engagement and persistent participation across time. There was an exchange of information and ideas primarily through a microblogging format but nimbly cross-linking to platforms with more knowledge depth, such as blogs and media hubs (eg, YouTube and Vimeo). The community shows a topology similar to other mesh-organized networks, in particular high centrality hubs or users and content.

The main challenge of a digital community of people with little interaction in real life is translating the information, knowledge, and innovation into practice change [35]. However, the #FOAMed community is able to generate content, refine its applicability, and identify tailored and meaningful adaptations based on the information negotiated. This is evidenced in the sentiment analysis of the content, as well as the organization around specific links. It appears that some members of the group serve as knowledge gatekeepers without forcing the community’s focus (demonstrated by the significance of the links and by the concentrated domains where information is stored and appraised). In terms of evidence of real-world application of the knowledge created, analysis of the content of the information suggests they are actionable concepts; however, a proof of application in real practice is difficult to obtain using an online analysis and is a limitation of this study. The conversations held in the forum of the comments sections of the most commonly shared blogs showed the social construct of testing, challenging, and contextualizing new knowledge, and they challenged assumptions of the different clinical practice areas of the contributors. These interactions ranged from affirmations agreeing that the new knowledge was practice changing, to instances of reflection that changes had been made with positive results. We observed a process of affirmation, contextualization to current or changed practice, and presentation of further evidence by way of linking to other primary or secondary literature on the discussed topic.

### Limitations

A major limitation of our study is that the fine detail interlinking community practice has not been confirmed. This will require a different methodological approach, which is not possible in the scope of this study. Given the clear matching of the dataset to the a priori postulate, further more detailed work seems worthwhile.

Further limitations to this work include the inability to confirm alterations in day-to-day patient clinical outcomes by network participants. However, the size of the network and its continued expansion and evolution suggest engagement through an evidence hierarchy that reaches beyond perceived or reported benefit. We believe the main implication of this preliminary evidence of an oCoP is the possible adoption of this structure (ie, a decentralized, distributed, self-regulated, diverse, nonhierarchical network of users and knowledge) as a suitable, relatively cheap and powerful method for knowledge management in health care teams and institutions.

The emergence of an oCoP oriented toward collaboration and the creation, curation, appraisal, and dissemination of knowledge would be a paradigm change in medical education. The concept of education based on a CoP have been described previously [11], but its existence with regard to medical education has never been shown before, although theoretical benefits have been considered and anxiously anticipated [9,36], with some authors arguing that social learning may replace formal training [9].

### Conclusion

The advent of a network devoted to knowledge translation constitutes a new frontier for health care education and knowledge management, with consequences that we are just beginning to understand. Our work shows that social media have been innovative in progressing medical education and that identification of oCoPs within social media may well be possible. This new framework of knowledge organization may appear as a new model for the management of information and users in a network-based health care system.

## Appendix

Multimedia Appendix 1 Top 100 influencers in the #FOAMed community.

Multimedia Appendix 2 Weighted network centrality metrics, based on mentions, hub, and authority quotients.

Multimedia Appendix 3 Top 150 conversations generated by the #FOAMed community.

Multimedia Appendix 4 Top 100 links shared by the #FOAMed community.

Multimedia Appendix 5 Netnographic evaluation of digital products from #FOAMed conversations.

## Abbreviations

CoP: community of practice
FOAM: free open access medical education
oCoP: online community of practice
