# Supplementary material for: Preliminary Evidence for the Emergence of a Health Care Online Community of Practice: Using a Netnographic Framework for Twitter Hashtag Analytics
Source: J Med Internet Res. 2017 Jul 14;19(7):e252. doi: 10.2196/jmir.7072 (PMC5533942; doi:10.2196/jmir.7072)
Supplement: Multimedia Appendix 1 [file jmir_v19i7e252_app1.pdf]

Top 100 Influencers, Stratified by Number of Mentions

Mentions	User Handle	Name	Health Care Role	Followers, No.
26,140	@srrezaie	Salim R. Rezaie	Doctor	5,988
10,494	@emswami	Anand Swaminathan	Doctor	7,516
5,278	@crit_care	Critical Care	Org other health care	5,391
5,258	@foam_highlights	FOAM Highlights	Org other health care	13,286
5,086	@em_resus	Sam Ghali	Doctor	2,128
5,016	@ketaminh	Minh Le Cong	Doctor	10,010
4,982	@ems12lead	EMS 12-Lead	Org other health care	13,053
4,946	@nfpineda	Nicolas Pineda	Doctor	601
4,943	@radiopaedia	Radiopaedia.org	Org other health care	16,085
4,909	@precordialthump	Chris Nickson	Doctor	11,517
4,809	@smithecgblog	Stephen W. Smith	Doctor	6,094
4,338	@cabreraerdr	Daniel Cabrera	Doctor	1,962
4,309	@emcrit	Scott Weingart	Doctor	21,375
4,300	@thesgem	Ken Milne	Doctor	3,589
4,240	@emlitofnote	Ryan Radecki	Doctor	5,814
4,178	@ultrasoundpod	Matt and Mike	Org other health care	7,967
4,124	@_nmay	Natalie May	Doctor	5,148
4,070	@radiologysigns	Radiology Signs	Org other health care	17,755
3,839	@sandnsurf	Mike Cadogan	Doctor	17,392
3,584	@lwestafer	Lauren Westafer	Doctor	3,692
3,415	@joelex5	Joe Lex	Doctor	5,765
3,293	@andyneill	Andy Neill	Doctor	4,829
3,215	@em_educator	rob rogers	Doctor	8,302
3,141	@mayoclinicem	Mayo Clinic EM	Org provider	2,175
3,059	@njoshi8	Nikita Joshi	Researcher/ academic	2,533
2,965	@amalmattu	Amal Mattu	Doctor	14,418
2,940	@pedemmorsels	Sean M. Fox	Doctor	2,250
2,932	@knowmedge	knowmedge	Org other health care	10,319
2,854	@pharmertoxguy	Bryan D. Hayes	HCP	7,569
2,834	@emmanchester	Simon Carley	Doctor	8,224
2,777	@bedsidesono	Mike Stone	Doctor	3,863

2,720	@emdocsdotnet	emdocs team	Org advocate/ supporter	1,214
2,683	@criticalcarenow	Haney Mallemat	Doctor	10,269
2,651	@stemlyns	Saint Emlyn’s	Org other health care	5,515
2,557	@ultrasoundjelly	Ben C. Smith	Doctor	1,569
2,554	@reeldx	ReelDx	Org other non− health care	3,668
2,541	@clinicalcaserev	Clinical Case Review	Org advocate/ supporter	18,787
2,397	@mastinmd	Matt Astin	Doctor	851
2,330	@jeremyfaust	jeremy faust	Doctor	3,831
2,306	@kangaroobeach	Tim Leeuwenburg	Doctor	4,640
2,276	@m_lin	Michelle Lin	Doctor	11,498
2,247	@brent_thoma	Brent Thoma	Doctor	3,647
2,205	@jama_current	JAMA	Org other health care	136,714
2,160	@rcemfoamed	RCEM FOAMed Network	Org research/ academic	4,227
2,110	@broomedocs	Casey Parker	Doctor	4,275
2,104	@cmcem	Carolinas Med Ctr EM	Org provider	664
2,066	@boringem	BoringEM	Org advocate/ supporter	2,547
2,041	@ultrasoundmd	Jacob Avila	Doctor	1,385
2,038	@wicsbottomline	The Bottom Line	Org advocate/ supporter	3,138
2,014	@nejm	NEJM	Org other health care	205,624
1,948	@ecgtraining	ECG Medical Training	Org advocate/ supporter	1,827
1,941	@mededucation101	MedEd101	HCP	6,164
1,899	@tessardavis	Tessa Davis	Doctor	2,071
1,893	@umanamd	Manrique Umana	Doctor	2,902
1,889	@tchanmd	Teresa Chan	Doctor	3,587
1,883	@david_reeldx	David Spiro MD, MPH	Doctor	21,172
1,822	@painfreeed	Sergey Motov	Doctor	2,093
1,707	@airwaycam	Richard Levitan	Doctor	3,467
1,695	@emhighak	Alex Koyfman	Doctor	773
1,692	@mdaware	Seth Trueger	Doctor	8,193
1,568	@foampodcast	FOAMcast	Org advocate/ supporter	2,938

1,524	@cliffreid	Cliff Reid	Individual other non−health care	9,559
1,459	@smaccteam	The SMACC Team	Org advocate/ supporter	10,473
1,437	@pulmcrit	Josh Farkas	Doctor	1,452
1,406	@emcases	Anton Helman	Doctor	2,336
1,366	@tamingthesru	UC EM Residency	Org research/ academic	1,788
1,345	@davidjuurlink	David Juurlink	HCP	7,754
1,340	@mue_14	MUE_USS	Org research/ academic	570
1,327	@templeem	Temple EM Residency	Org provider	1,898
1,325	@emnerd_	Rory Spiegel	Doctor	1,357
1,276	@petrosoniak	Andrew Petrosoniak	Doctor	1,117
1,262	@amermedicalassn	AMA	Org other health care	377,423
1,256	@usmleaid	USMLE AID	Org advocate/ supporter	16,332
1,232	@drmarkusalanis	Markus Alanis	Doctor	1,720
1,225	@eusmd	Jason Fischer	Individual other health care	706
1,224	@damian_roland	Damian Roland	Doctor	4,871
1,190	@ucirvineem	UC Irvine EM	Org research/ academic	716
1,173	@learntheheart	LearnTheHeart.com	Org advocate/ supporter	17,974
1,152	@iceman_ex	Olusegun Olusanya	Doctor	1,046
1,146	@poisonreview	Leon Gussow	Doctor	4,612
1,096	@inject_orange	Jesse Spurr	HCP	2,085
1,060	@wustl_em	WashU EM Residency	Org research/ academic	793
1,051	@aliemteam	Academic Life in EM	Org advocate/ supporter	3,640
1,036	@hansvanschuppen	Hans van Schuppen	Doctor	1,727
1,025	@emimdoc	David Marcus	Doctor	3,801
1,008	@templeemus	Temple EM Ultrasound	Org provider	976
1,007	@emnews	EmergncyMedicineNews	Org other health care	13,214
984	@sonospot	Laleh Gharahbaghian	Doctor	2,169
964	@mfbellolio	Fernanda Bellolio	Doctor	820
963	@embasic	Steve Carroll, DO	Doctor	6,465
955	@fltdoc1	Mike Abernethy	Doctor	3,416

929	@purdy_eve	Eve Purdy	Individual other health care	1,810
920	@core_em	CoreEM	Org research/ academic	1,108
919	@socraticem	Victoria Brazil	Doctor	3,113
910	@annalsofem	AnnalsofEM	Org advocate/ supporter	7,224
867	@johngreenwoodmd	John Greenwood	Doctor	1,970
861	@docbond007	Michael Bond, MD	Doctor	1,566
840	@emeducation	Rob Cooney, MD, MEd	Doctor	3,883
829	@urgenciauc	Urgencia UC	Org research/ academic	788
823	@heftemcast	HEFT EMCAST	Org advocate/ supporter	1,928

Abbreviations: HCP, health care professionals; Org, organization.
