# Supplementary material for: Preliminary Evidence for the Emergence of a Health Care Online Community of Practice: Using a Netnographic Framework for Twitter Hashtag Analytics
Source: J Med Internet Res. 2017 Jul 14;19(7):e252. doi: 10.2196/jmir.7072 (PMC5533942; doi:10.2196/jmir.7072)
Supplement: Multimedia Appendix 2 [file jmir_v19i7e252_app2.pdf]

Weighted network centrality metrics, based on mentions, hub, and authority quotients.

User	Weight	Edges (links)	Hub	Authority
Salim R. Rezaie	25	149	2.68×10^{-3}	5.67×10^{-3}
Anand Swaminathan	10	77	2.80×10^{-3}	4.78×10^{-3}
Critical Care	6	23	2.50×10^{-3}	4.48×10^{-3}
FOAM Highlights	6	9	0	4.34×10^{-3}
Radiopaedia.org	5	22	2.50×10^{-3}	4.47×10^{-3}
Chris Nickson	5	27	2.57×10^{-3}	4.47×10^{-3}
Minh Le Cong	5	64	2.99×10^{-3}	4.44×10^{-3}
Ken Milne	5	23	2.57×10^{-3}	4.42×10^{-3}
Nicolas Pineda	5	27	2.61×10^{-3}	4.42×10^{-3}
EMS 12-Lead	5	19	2.54×10^{-3}	4.40×10^{-3}
Sam Ghali	5	13	0	4.39×10^{-3}
Stephen W. Smith	5	12	0	4.38×10^{-3}
Scott Weingart	5	10	0	4.36×10^{-3}
Daniel Cabrera	5	33	2.74×10^{-3}	4.35×10^{-3}
Matt and Mike	4	21	2.53×10^{-3}	4.43×10^{-3}
Joe Lex	4	16	2.50×10^{-3}	4.41×10^{-3}
Radiology Signs	4	16	2.50×10^{-3}	4.40×10^{-3}
Lauren Westafer	4	14	2.52×10^{-3}	4.37×10^{-3}
Natalie May	4	18	2.58×10^{-3}	4.36×10^{-3}
Ryan Radecki	4	12	2.52×10^{-3}	4.35×10^{-3}
Mike Cadogan	4	7	0	4.32×10^{-3}
Andy Neill	4	1	0	4.26×10^{-3}
Clinical Case Review	3	24	2.51×10^{-3}	4.48×10^{-3}
rob rogers	3	19	2.52×10^{-3}	4.43×10^{-3}
Sean M. Fox	3	14	2.50×10^{-3}	4.39×10^{-3}
Nikita Joshi	3	23	2.60×10^{-3}	4.39×10^{-3}
Mike Stone	3	13	0	4.39×10^{-3}
Matt Astin	3	14	2.52×10^{-3}	4.38×10^{-3}
Amal Mattu	3	11	0	4.37×10^{-3}
Mayo Clinic EM	3	16	2.57×10^{-3}	4.34×10^{-3}
Haney Mallemat	3	10	2.51×10^{-3}	4.34×10^{-3}
emdocs team	3	8	0	4.33×10^{-3}
RCEM FOAMed Network	3	8	0	4.33×10^{-3}
knowmedge	3	10	2.53×10^{-3}	4.32×10^{-3}
Michelle Lin	3	6	0	4.31×10^{-3}
Simon Carley	3	8	2.51×10^{-3}	4.31×10^{-3}
Bryan D. Hayes	3	6	0	4.31×10^{-3}
Ben C. Smith	3	8	2.51×10^{-3}	4.31×10^{-3}
Saint Emlyn’s	3	6	2.50×10^{-3}	4.30×10^{-3}
jeremy faust	3	8	2.52×10^{-3}	4.30×10^{-3}
ReelDx	3	10	2.54×10^{-3}	4.30×10^{-3}
Tim Leeuwenburg	3	6	2.52×10^{-3}	4.28×10^{-3}
The Bottom Line	2	9	0	4.34×10^{-3}
MedEd101	2	9	2.50×10^{-3}	4.33×10^{-3}
MUE_USS	2	13	2.56×10^{-3}	4.32×10^{-3}

Jason Fischer	2	11	$2.53{\times}10^{-3}$	$4.32{\times}10^{-3}$
Casey Parker	2	14	$2.58{\times}10^{-3}$	$4.31{\times}10^{-3}$
Brent Thoma	2	15	$2.59{\times}10^{-3}$	$4.31{\times}10^{-3}$
Tessa Davis	2	6	$2.50{\times}10^{-3}$	$4.30{\times}10^{-3}$
UC EM Residency	2	9	$2.53{\times}10^{-3}$	$4.30{\times}10^{-3}$
Carolinas Med Ctr EM	2	7	$2.51{\times}10^{-3}$	$4.30{\times}10^{-3}$
Jacob Avila	2	8	$2.52{\times}10^{-3}$	$4.30{\times}10^{-3}$
ECG Medical Training	2	6	$2.50{\times}10^{-3}$	$4.30{\times}10^{-3}$
David Spiro MD, MPH	2	8	$2.52{\times}10^{-3}$	$4.30{\times}10^{-3}$
Temple EM Residency	2	5	0	$4.30{\times}10^{-3}$
UC Irvine EM	2	5	0	$4.30{\times}10^{-3}$
Richard Levitan	2	4	0	$4.30{\times}10^{-3}$
Teresa Chan	2	18	$2.65{\times}10^{-3}$	$4.29{\times}10^{-3}$
Seth Trueger	2	10	$2.57{\times}10^{-3}$	$4.28{\times}10^{-3}$
Olusegun Olusanya	2	16	$2.64{\times}10^{-3}$	$4.28{\times}10^{-3}$
Andrew Petrosoniak	2	4	$2.50{\times}10^{-3}$	$4.28{\times}10^{-3}$
Alex Koyfman	2	16	$2.64{\times}10^{-3}$	$4.28{\times}10^{-3}$
BoringEM	2	6	$2.52{\times}10^{-3}$	$4.28{\times}10^{-3}$
Manrique Umana	2	16	$2.65{\times}10^{-3}$	$4.27{\times}10^{-3}$
Rory Spiegel	2	7	$2.55{\times}10^{-3}$	$4.27{\times}10^{-3}$
Sergey Motov	2	21	$2.70{\times}10^{-3}$	$4.27{\times}10^{-3}$
FOAMcast	2	3	$2.50{\times}10^{-3}$	$4.27{\times}10^{-3}$
The SMACC Team	2	3	$2.51{\times}10^{-3}$	$4.26{\times}10^{-3}$
Cliff Reid	2	1	0	$4.26{\times}10^{-3}$
Damian Roland	2	3	$2.51{\times}10^{-3}$	$4.26{\times}10^{-3}$
Leon Gussow	2	2	$2.51{\times}10^{-3}$	0
Anton Helman	2	1	$2.51{\times}10^{-3}$	0
Victoria Brazil	1	8	0	$4.33{\times}10^{-3}$
Urgencia UC	1	8	$2.50{\times}10^{-3}$	$4.32{\times}10^{-3}$
Salim R. Rezaie	1	6	0	$4.31{\times}10^{-3}$
Laleh Gharahbaghian	1	7	$2.51{\times}10^{-3}$	$4.30{\times}10^{-3}$
terrence mulligan	1	5	0	$4.30{\times}10^{-3}$
Eve Purdy	1	9	$2.55{\times}10^{-3}$	$4.29{\times}10^{-3}$
Temple EM Ultrasound	1	6	$2.51{\times}10^{-3}$	$4.29{\times}10^{-3}$
Egerton Y Davis IV	1	3	0	$4.28{\times}10^{-3}$
Emergency Cardiac	1	5	$2.51{\times}10^{-3}$	$4.28{\times}10^{-3}$
EMSWorld News	1	3	0	$4.28{\times}10^{-3}$
Resa E Lewiss	1	12	$2.59{\times}10^{-3}$	$4.28{\times}10^{-3}$
Sergio Alvizu	1	6	$2.52{\times}10^{-3}$	$4.28{\times}10^{-3}$
Clinical Monster	1	3	0	$4.28{\times}10^{-3}$
Mark Tessaro	1	5	$2.51{\times}10^{-3}$	$4.28{\times}10^{-3}$
Charisse Kwan, MD	1	6	$2.52{\times}10^{-3}$	$4.28{\times}10^{-3}$
EM:RAP’s Mel Herbert	1	2	0	$4.27{\times}10^{-3}$
Penny Wilson	1	3	$2.50{\times}10^{-3}$	$4.27{\times}10^{-3}$
reuben strayer	1	2	0	$4.27{\times}0^{-3}$
EMS Innovation	1	2	0	$4.27{\times}10^{-3}$
Fernanda Bellolio	1	5	$2.52{\times}10^{-3}$	$4.27{\times}10^{-3}$
Dustin Leigh, MD	1	3	$2.50{\times}10^{-3}$	$4.27{\times}10^{-3}$
Cameron Wangsgard	1	2	0	$4.27{\times}10^{-3}$
Tom Bouthillet	1	9	$2.57{\times}10^{-3}$	$4.27{\times}10^{-3}$

Cian O’Brien	1	2	0	4.27×10^{-3}
Alan Batt	1	2	0	4.27×10^{-3}
WashU EM Residency	1	4	2.51×10^{-3}	4.27×10^{-3}
Word on the Street	1	3	2.50×10^{-3}	4.27×10^{-3}
christopher picard	1	2	0	4.27×10^{-3}
