# Supplementary material for: Preliminary Evidence for the Emergence of a Health Care Online Community of Practice: Using a Netnographic Framework for Twitter Hashtag Analytics
Source: J Med Internet Res. 2017 Jul 14;19(7):e252. doi: 10.2196/jmir.7072 (PMC5533942; doi:10.2196/jmir.7072)
Supplement: Multimedia Appendix 3 [file jmir_v19i7e252_app3.pdf]

Top 150 Conversation Threads Showing a Branching Structure

Thread	Branch	User	Reply To
1	1	@srrezaie	
	1.1	@pedemmorsels	@srrezaie
	1.2	@theredone1977	@srrezaie
	1.3	@paramedickiwi	@srrezaie
	1.4	@vinpel	@srrezaie
	1.5	@jsnyder_dpt	@srrezaie
	1.5.1	@kulturfizikci	@jsnyder_dpt
	1.6	@medclerkships	@srrezaie
	1.7	@lore_cad	@srrezaie
	1.8	@ivline	@srrezaie
	1.9	@zambezi40	@srrezaie
	1.1	@polythenia	@srrezaie
	1.11	@gpwhyte	@srrezaie
	1.12	@ajpsportsrd	@srrezaie
	1.13	@Trevail	@srrezaie
	1.14	@kpsdfmr	@srrezaie
	1.15	@marmaraacil	@srrezaie
2	2	@ketaminh	
	2.1	@agosbell	@ketaminh
	2.1.1	@ketaminh	@agosbell
	2.1.1.1	@agosbell	@ketaminh
	2.2	@jon_kavanagh	@ketaminh
	2.3	@_nmay	@ketaminh
	2.4	@kangaroobeach	@ketaminh
	2.4.1	@nicjhig	@kangaroobeach
	2.5	@inject_orange	@ketaminh
	2.6	@damian_roland	@ketaminh
	2.6.1	@damian_roland	@damian_roland
	2.7	@spaynesarah	@ketaminh
	2.8	@sjwriteshere	@ketaminh
	2.9	@iceman_ex	@ketaminh
3	3	@jsandmd	
	3.1	@jsandmd	@jsandmd
	3.2	@jsandmd	@jsandmd
	3.3	@jsandmd	@jsandmd
	3.3.1	@embasic	@jsandmd
	3.3.2	@ivline	@jsandmd
	3.3.3	@ivline	@jsandmd
	3.3.3.1	@ivline	@ivline

	3.4	@jsandmd	@jsandmd
	3.5	@jsandmd	@jsandmd
	3.6	@jsandmd	@jsandmd
	3.7	@jsandmd	@jsandmd
	3.8	@jsandmd	@jsandmd
	3.9	@lainieyarris	@jsandmd
4	4	@sandnsurf	
	4.1	@agosbell	@sandnsurf
	4.2	@kirstychallen	@sandnsurf
	4.3	@georginarosos	@sandnsurf
	4.4	@thomas1973	@sandnsurf
	4.5	@stuart_duffin	@sandnsurf
	4.6	@medwithaltitude	@sandnsurf
	4.7	@_nmay	@sandnsurf
	4.8	@umanamd	@sandnsurf
	4.9	@emurgentologist	@sandnsurf
	4.1	@mdaware	@sandnsurf
5	5	@welshgasdoc	
	5.1	@emilyflump	@welshgasdoc
	5.2	@mcarpenter1967	@welshgasdoc
	5.3	@drannemurphy	@welshgasdoc
	5.4	@jennenzie	@welshgasdoc
	5.5	@docrickywood	@welshgasdoc
	5.6	@sstirling1980	@welshgasdoc
	5.7	@strictlykaren	@welshgasdoc
	5.8	@pennyl50	@welshgasdoc
	5.9	@janmdavies	@welshgasdoc
6	6	@richardbody	
	6.1	@kangaroobeach	@richardbody
	6.2	@andywebster	@richardbody
	6.2.1	@andywebster	@andywebster
	6.3	@andywebster	@richardbody
	6.4	@tonyjoy81	@richardbody
	6.5	@alittlemedic	@richardbody
	6.6	@cathtaylorpef	@richardbody
	6.7	@damian_roland	@richardbody
	6.8	@10emconf	@richardbody
7	7	@thesgem	
	7.1	@choo_ek	@thesgem
	7.2	@felixankel	@thesgem
	7.2.1	@thesgem	@felixankel

	7.4	@mrvanbalken	@thesgem
	7.5	@drwaisun	@thesgem
	7.6	@mkchan_rcpsc	@thesgem
	7.7	@respirologist	@thesgem
8	8	@foameduk	
	8.1	@foameduk	@foameduk
	8.1.1	@foameduk	@foameduk
	8.1.1.1	@foameduk	@foameduk
	8.1.1.2	@foameduk	@foameduk
	8.1.1.2.1	@foameduk	@foameduk
	8.1.1.2.1.1	@foameduk	@foameduk
	8.1.1.2.1.1.1	@foameduk	@foameduk
	8.1.1.2.1.1.1.1	@foameduk	@foameduk
9	9	@clinicalcaserev	
	9.1	@harleenwray	@clinicalcaserev
	9.2	@dannysafarimd	@clinicalcaserev
	9.3	@clinicalcaserev	@clinicalcaserev
	9.3.1	@allenwellmd	@clinicalcaserev
	9.3.2	@larizamarz25	@clinicalcaserev
	9.3.2.1	@isaacaladeh	@larizamarz25
	9.3.3	@aldicarlom	@clinicalcaserev
	9.3.4	@drakates	@clinicalcaserev
10	10	@ausnurseed	
	10.1	@kangaroobeach	@ausnurseed
	10.1.1	@paramedrusty	@kangaroobeach
	10.1.2	@inject_orange	@kangaroobeach
	10.2	@kangaroobeach	@ausnurseed
	10.3	@andrewacnp1	@ausnurseed
	10.3.1	@thenursepath	@andrewacnp1
	10.3.2	@thenursepath	@andrewacnp1
11	11	@em_educator	
	11.1	@emimdoc	@em_educator
	11.1.1	@johnboy237	@emimdoc
	11.1.1.1	@emimdoc	@johnboy237
	11.1.1.1.1	@johnboy237	@emimdoc
	11.1.1.1.1.1	@emimdoc	@johnboy237
	11.1.2	@emmanchester	@emimdoc
12	12	@nomadicgp	
	12.1	@embasic	@nomadicgp
	12.2	@doconskis	@nomadicgp
	12.3	@doconskis	@nomadicgp

	12.4	@doconskis	@nomadicgp
	12.5	@kirstychallen	@nomadicgp
	12.6	@kangaroobeach	@nomadicgp
13	13	@radiopaedia	
	13.1	@cjpoynter	@radiopaedia
	13.2	@heatherm211	@radiopaedia
	13.2.1	@thinkalot	@heatherm211
	13.3	@philipcosson	@radiopaedia
	13.4	@heartotxheartmd	@radiopaedia
	13.5	@voight21	@radiopaedia
14	14	@apmeded	
	14.1	@tamingthesru	@apmeded
	14.2	@idiottracker	@apmeded
	14.3	@idiottracker	@apmeded
	14.4	@idiottracker	@apmeded
	14.4.1	@apmeded	@idiottracker
	14.5	@idiottracker	@apmeded
15	15	@emcapetown	
	15.1	@emcapetown	@emcapetown
	15.2	@emcapetown	@emcapetown
	15.3	@emcapetown	@emcapetown
	15.4	@emcapetown	@emcapetown
	15.5	@emcapetown	@emcapetown
	15.6	@emcapetown	@emcapetown
16	16	@clinicalcaserev	
	16.1	@larizamarz25	@clinicalcaserev
	16.2	@andresvitarmd	@clinicalcaserev
	16.3	@clinicalcaserev	@clinicalcaserev
	16.4	@clinicalcaserev	@clinicalcaserev
	16.4.1	@sophiaperlick	@clinicalcaserev
17	17	@clinicalcaserev	
	17.1	@clinicalcaserev	@clinicalcaserev
	17.2	@andresvitarmd	@clinicalcaserev
	17.3	@clinicalcaserev	@clinicalcaserev
	17.3.1	@david_reeldx	@clinicalcaserev
	17.3.2	@dralizduarte	@clinicalcaserev
18	18	@elderlymeded	
	18.1	@elderlymeded	@elderlymeded
	18.1.1	@amcunningham	@elderlymeded
	18.1.1.1	@elderlymeded	@amcunningham
	18.1.1.2	@elderlymeded	@amcunningham

	18.2	@beachdadair	@elderlymeded
19	19	@clinicalcaserev	
	19.1	@clinicalcaserev	@clinicalcaserev
	19.1.1	@dominicanyummd	@clinicalcaserev
	19.2	@clinicalcaserev	@clinicalcaserev
	19.2.1	@sophiaperlick	@clinicalcaserev
	19.3	@drakates	@clinicalcaserev
20	20	@ruralgreengp	
	20.1	@kangaroobeach	@ruralgreengp
	20.2	@nomadicgp	@ruralgreengp
	20.3	@kangaroobeach	@ruralgreengp
	20.3.1	@ruralgreengp	@kangaroobeach
	20.3.1.1	@kangaroobeach	@ruralgreengp
21	21	@brent_thoma	
	21.1	@andrewbuelt	@brent_thoma
	21.2	@perronimd	@brent_thoma
	21.3	@medclerkships	@brent_thoma
	21.4	@lochie561	@brent_thoma
	21.5	@inject_orange	@brent_thoma
22	22	@sandnsurf	
	22.1	@tchanmd	@sandnsurf
	22.2	@louaud	@sandnsurf
	22.3	@stemlyns	@sandnsurf
	22.4	@lucyhindle1	@sandnsurf
	22.5	@surferkirst	@sandnsurf
23	23	@ems_junkie	
	23.1	@brent_thoma	@ems_junkie
	23.1.1	@ems_junkie	@brent_thoma
	23.1.1.1	@brent_thoma	@ems_junkie
	23.1.1.1.1	@brent_thoma	@brent_thoma
	23.1.1.2	@pharmertoxguy	@ems_junkie
24	24	@emsavenger	
	24.1	@bobbyboop86	@emsavenger
	24.1.1	@emsavenger	@bobbyboop86
	24.1.1.1	@bobbyboop86	@emsavenger
	24.1.1.2	@bobbyboop86	@emsavenger
	24.1.1.3	@bobbyboop86	@emsavenger
25	25	@em_resus	
	25.1	@bellabertie	@em_resus
	25.2	@ginger_hart	@em_resus
	25.3	@megpearlz	@em_resus

	25.4	@serkane_eroglu	@em_resus
	25.5	@courtlarkin	@em_resus
26	26	@joelex5	
	26.1	@dpt2go	@joelex5
	26.2	@mikedavis8702	@joelex5
	26.3	@jendlake	@joelex5
	26.4	@vivaphysicians	@joelex5
	26.4.1	@drannealbers	@vivaphysicians
27	27	@knowmedge	
	27.1	@larizamarz25	@knowmedge
	27.2	@marysandum	@knowmedge
	27.3	@clinicalcaserev	@knowmedge
	27.3.1	@allenwellmd	@clinicalcaserev
	27.4	@aldicarlom	@knowmedge
28	28	@lonerganco	
	28.1	@pennyatkinson72	@lonerganco
	28.2	@jonathanhearsey	@lonerganco
	28.3	@MattTyrermusic	@lonerganco
	28.4	@gruoch	@lonerganco
	28.5	@drgrovermd	@lonerganco
29	29	@dralangrayson	
	29.1	@ketaminh	@dralangrayson
	29.2	@alexwintermute	@dralangrayson
	29.3	@andrewbuelt	@dralangrayson
	29.4	@keirshiels	@dralangrayson
	29.5	@diverselearners	@dralangrayson
30	30	@nomadicgp	@lwestafer
	30.1	@nomadicgp	@nomadicgp
	30.1.1	@kangaroobeach	@nomadicgp
	30.2	@hotsahs	@nomadicgp
	30.3	@mdaware	@nomadicgp
31	31	@s_p_md	
	31.1	@venkbellamkonda	@s_p_md
	31.1.1	@s_p_md	@venkbellamkonda
	31.1.2	@s_p_md	@venkbellamkonda
	31.2	@venkbellamkonda	@s_p_md
32	32	@emmanchester	
	32.1	@drjimblackburn	@emmanchester
	32.2	@criticalmusing	@emmanchester
	32.3	@hannahpopsy	@emmanchester
	32.4	@jrshorthouse	@emmanchester

33	33	@pedemmorsels	
	33.1	@damian_roland	@pedemmorsels
	33.1.1	@pedemmorsels	@damian_roland
	33.1.2	@pedemmorsels	@damian_roland
	33.1.2.1	@damian_roland	@pedemmorsels
34	34	@clinicalcaserev	
	34.1	@dominicanyummd	@clinicalcaserev
	34.2	@isaacaladeh	@clinicalcaserev
	34.3	@clinicalcaserev	@clinicalcaserev
	34.3.1	@sophiaperlick	@clinicalcaserev
35	35	@clinicalcaserev	
	35.1	@isaacaladeh	@clinicalcaserev
	35.2	@isaacaladeh	@clinicalcaserev
	35.3	@stefanoamir	@clinicalcaserev
	35.4	@peternmd	@clinicalcaserev
36	36	@pulmcrit	
	36.1	@cjchiu	@pulmcrit
	36.2	@cjchiu	@pulmcrit
	36.3	@dateswhitecoats	@pulmcrit
	36.4	@khanijomd	@pulmcrit
37	37	@clinicalcaserev	
	37.1	@onlydreamsss	@clinicalcaserev
	37.2	@dannysafarimd	@clinicalcaserev
	37.3	@drakates	@clinicalcaserev
	37.4	@drakates	@clinicalcaserev
38	38	@docbrent	
	38.1	@emmanchester	@docbrent
	38.1.1	@docbrent	@emmanchester
	38.2	@doc_ryan	@docbrent
	38.2.1	@docbrent	@doc_ryan
39	39	@njoshi8	
	39.1	@jsemple88	@njoshi8
	39.1.1	@njoshi8	@jsemple88
	39.2	@tachypnoeic	@njoshi8
	39.3	@umanamd	@njoshi8
40	40	@templeemus	
	40.1	@templeemus	@templeemus
	40.1.1	@templeemus	@templeemus
	40.1.2	@templeemus	@templeemus
	40.2	@templeemus	@templeemus
41	41	@karimbrohi	

	41.1	@thomas1973	@karimbrohi
	41.2	@kirstychallen	@karimbrohi
	41.3	@pcjawick	@karimbrohi
	41.4	@jstgeorgemd	@karimbrohi
42	42	@em_educator	
	42.1	@fattriman	@em_educator
	42.2	@vcrodrigues_uea	@em_educator
	42.3	@rafaeltimermans	@em_educator
	42.4	@maytemoren	@em_educator
43	43	@srrezaie	
	43.1	@heatherm211	@srrezaie
	43.2	@carrings	@srrezaie
	43.3	@ilindelatorremd	@srrezaie
	43.4	@ilindelatorremd	@srrezaie
44	44	@_nmay	
	44.1	@docshannon	@_nmay
	44.2	@saspist	@_nmay
	44.3	@physiorichmond	@_nmay
	44.4	@maxirebecca	@_nmay
45	45	@aysabbagh	
	45.1	@aysabbagh	@aysabbagh
	45.1.1	@aysabbagh	@aysabbagh
	45.1.1.1	@aysabbagh	@aysabbagh
	45.1.1.2	@aysabbagh	@aysabbagh
46	46	@kangaroobeach	
	46.1	@broomedocs	@kangaroobeach
	46.1.1	@davidrhogg	@broomedocs
	46.1.1.1	@broomedocs	@davidrhogg
	46.1.2	@alittlemedic	@broomedocs
47	47	@clinicalcaserev	
	47.1	@clinicalcaserev	@clinicalcaserev
	47.1.1	@maini_jr	@clinicalcaserev
	47.1.2	@dralizduarte	@clinicalcaserev
	47.1.3	@dominicanyummd	@clinicalcaserev
48	48	@precordialthump	
	48.1	@thetopend	@precordialthump
	48.2	@lwestafer	@precordialthump
	48.3	@vinpel	@precordialthump
	48.4	@joncheah	@precordialthump
49	49	@alkhalifaa2	
	49.1	@alkhalifaa2	@alkhalifaa2

	49.1.1	@alkhalifaa2	@alkhalifaa2
	49.1.2	@alkhalifaa2	@alkhalifaa2
	49.2	@usmlestepprep	@alkhalifaa2
50	50	@clinicalcaserev	
	50.1	@marysandum	@clinicalcaserev
	50.2	@harleenwray	@clinicalcaserev
	50.3	@clinicalcaserev	@clinicalcaserev
	50.4	@clinicalcaserev	@clinicalcaserev
51	51	@smithecgblog	
	51.1	@apathetic_cynic	@smithecgblog
	51.2	@ecctrainings	@smithecgblog
	51.3	@ertcenter	@smithecgblog
	51.4	@gflores911	@smithecgblog
52	52	@clinicalcaserev	
	52.1	@clinicalcaserev	@clinicalcaserev
	52.1.1	@sophiaperlick	@clinicalcaserev
	52.1.2	@alhasilnan	@clinicalcaserev
	52.2	@andresvitarmd	@clinicalcaserev
	53.1	@dasabuvir	@brodalumab
	53.2	@tocilizumab	@brodalumab
	53.2.1	@ixekizumab	@tocilizumab
	53.3	@obinutuzumab	@brodalumab
54	54	@lwestafer	
	54.1	@thesgem	@lwestafer
	54.1.1	@srrezaie	@thesgem
	54.1.2	@lwestafer	@thesgem
	54.1.2.1	@thesgem	@lwestafer
55	55	@pharmertoxguy	
	55.1	@global_em	@pharmertoxguy
	55.2	@global_em	@pharmertoxguy
	55.3	@drlaragoldstein	@pharmertoxguy
	55.4	@cathimon	@pharmertoxguy
56	56	@davidjuurlink	
	56.1	@bcm_emig	@davidjuurlink
	56.2	@drwaisun	@davidjuurlink
	56.3	@heartotxheartmd	@davidjuurlink
	56.4	@martinamcgowan	@davidjuurlink
57	57	@ermentor	
	57.1	@drdanica	@ermentor
	57.2	@drdanica	@ermentor
	57.2.1	@ermentor	@drdanica

	57.2.1.1	@drdanica	@ermentor
58	58	@_drjeffy	
	58.1	@njoshi8	@_drjeffy
	58.2	@_drjeffy	@_drjeffy
	58.2.1	@_drjeffy	@_drjeffy
	58.2.2	@ucaircaredoc	@_drjeffy
59	59	@clinicalcaserev	
	59.1	@stefanoamir	@clinicalcaserev
	59.2	@peternmd	@clinicalcaserev
	59.3	@clinicalcaserev	@clinicalcaserev
	59.3.1	@dominicanyummd	@clinicalcaserev
60	60	@drgdh	
	60.1	@stemlyns	@drgdh
	60.1.1	@drgdh	@stemlyns
	60.1.1.1	@stemlyns	@drgdh
	60.1.2	@drgdh	@stemlyns
61	61	@foamnglow	
	61.1	@sararellano	@foamnglow
	61.2	@jordannbaez	@foamnglow
	61.3	@15_macey	@foamnglow
	61.4	@1ging3r	@foamnglow
62	62	@foamnglow	
	62.1	@jamestsmith46	@foamnglow
	62.2	@nicole_lexy	@foamnglow
	62.3	@lauren_truax	@foamnglow
	62.4	@kenziee_king	@foamnglow
63	63	@apousson	
	63.1	@apousson	@apousson
	63.1.1	@apousson	@apousson
	63.1.1.1	@apousson	@apousson
	63.1.1.2	@apousson	@apousson
64	64	@stemlyns	
	64.1	@eleytherius	@stemlyns
	64.1.1	@eleytherius	@eleytherius
	64.2	@_lojones	@stemlyns
	64.3	@lizziek101	@stemlyns
65	65	@socalexmd	
	65.1	@kangaroobeach	@socalexmd
	65.1.1	@broomedocs	@kangaroobeach
	65.2	@damian_roland	@socalexmd
	65.3	@docib	@socalexmd

66	66	@davidjuurlink	
	66.1	@shanxonline	@davidjuurlink
	66.2	@haymanbuwan	@davidjuurlink
	66.3	@medclerkships	@davidjuurlink
	66.4	@rb_cavalcanti	@davidjuurlink
67	67	@tchanmd	
	67.1	@boringem	@tchanmd
	67.1.1	@mkchan_rcpsc	@boringem
	67.1.2	@dralangrayson	@boringem
	67.2	@aliemconf	@tchanmd
68	68	@foamnglow	
	68.1	@mak_dre69	@foamnglow
	68.2	@xoxo_deshai	@foamnglow
	68.2.1	@falliewalliee	@xoxo_deshai
	68.3	@lauren_rearick	@foamnglow
69	69	@_nmay	
	69.1	@kangaroobeach	@_nmay
	69.2	@markwiddowfield	@_nmay
	69.3	@duncanshrew	@_nmay
	69.4	@medwithaltitude	@_nmay
70	70	@precordialthump	
	70.1	@patientstormdoc	@precordialthump
	70.1.1	@patientstormdoc	@patientstormdoc
	70.1.2	@patientstormdoc	@patientstormdoc
71	71	@gracie_leo	
	71.1	@_thezol	@gracie_leo
	71.2	@emmanchester	@gracie_leo
	71.3	@morganbally	@gracie_leo
72	72	@tbayedguy	
	72.1	@tbayedguy	@tbayedguy
	72.1.1	@tbayedguy	@tbayedguy
	72.2	@fibroanestesia	@tbayedguy
73	73	@amiguelem	
	73.1	@praems	@amiguelem
	73.2	@ecctrainings	@amiguelem
	73.3	@gflores911	@amiguelem
74	74	@foamnglow	
	74.1	@amandamilne13	@foamnglow
	74.2	@stephen78745	@foamnglow
	74.3	@_dredreey	@foamnglow
75	75	@themattmak	

	75.1	@irishparamedic_	@themattmak
	75.2	@mjslabbert	@themattmak
	75.3	@hrmorriss	@themattmak
76	76	@foampodcast	
	76.1	@carrings	@foampodcast
	76.1.1	@foampodcast	@carrings
	76.2	@tmoadel	@foampodcast
77	77	@welshgasdoc	
	77.1	@harrysmyth	@welshgasdoc
	77.2	@gazzamagic78	@welshgasdoc
	77.3	@harrysmyth	@welshgasdoc
78	78	@emmanchester	
	78.1	@ciansobrien	@emmanchester
	78.2	@kasiahamptonmd	@emmanchester
	78.3	@akutdok	@emmanchester
79	79	@robapark	
	79.1	@fly_texan	@robapark
	79.2	@ketaminh	@robapark
	79.2.1	@robapark	@ketaminh
80	80	@emswami	
	80.1	@davidlendrum	@emswami
	80.2	@emedcurious	@emswami
	80.3	@nic_gilbert1	@emswami
81	81	@nomadicgp	
	81.1	@broomedocs	@nomadicgp
	81.2	@ketaminh	@nomadicgp
	81.3	@ernchang	@nomadicgp
82	82	@lwestafer	
	82.1	@ketaminh	@lwestafer
	82.1.1	@iceman_ex	@ketaminh
	82.1.2	@mdaware	@ketaminh
83	83	@foamnglow	
	83.1	@savannnersss	@foamnglow
	83.2	@_skylarlogan	@foamnglow
	83.3	@miss_miriamm	@foamnglow
84	84	@pharmertoxguy	
	84.1	@_nmay	@pharmertoxguy
	84.1.1	@jojohaber	@_nmay
	84.2	@dinod00	@pharmertoxguy
85	85	@mfbellolio	
	85.1	@mfbellolio	@mfbellolio

	85.1.1	@mfbellolio	@mfbellolio
	85.1.2	@mfbellolio	@mfbellolio
86	86	@afjem	
	86.1	@afjem	@afjem
	86.2	@afjem	@afjem
	86.2.1	@tomp_sa	@afjem
87	87	@clinicalcaserev	
	87.1	@dralizduarte	@clinicalcaserev
	87.2	@clinicalcaserev	@clinicalcaserev
	87.3	@clinicalcaserev	@clinicalcaserev
88	88	@teach_institute	
	88.1	@drlaragoldstein	@teach_institute
	88.2	@bhanders	@teach_institute
	88.3	@bretpnelson	@teach_institute
89	89	@srrezaie	
	89.1	@paramedickiwi	@srrezaie
	89.2	@rsireid	@srrezaie
	89.3	@heartotxheartmd	@srrezaie
90	90	@srrezaie	
	90.1	@alsomali	@srrezaie
	90.2	@ilindelatorremd	@srrezaie
	90.3	@dr_lara	@srrezaie
91	91	@naomihabib	
	91.1	@tchanmd	@naomihabib
	91.2	@emhighak	@naomihabib
	91.3	@knowmedge	@naomihabib
92	92	@painfreeed	
	92.1	@dateswhitecoats	@painfreeed
	92.2	@em_stevemcguire	@painfreeed
	92.3	@mahmoud_427	@painfreeed
93	93	@fltdoc1	
	93.1	@racheltoriana	@fltdoc1
	93.2	@paramedickiwi	@fltdoc1
	93.3	@jrshorthouse	@fltdoc1
94	94	@jeremyfaust	
	94.1	@brent_thoma	@jeremyfaust
	94.2	@drbokerjoker	@jeremyfaust
	94.3	@edgarvlermamd	@jeremyfaust
95	95	@racsurgeons	
	95.1	@ozvascdoc	@racsurgeons
	95.2	@csl888	@racsurgeons

	95.2.1	@ozvascdoc	@csl888
96	96	@clinicalcaserev	
	96.1	@clinicalcaserev	@clinicalcaserev
	96.2	@clinicalcaserev	@clinicalcaserev
	96.3	@lizziemarie08	@clinicalcaserev
97	97	@holleratbianca	
	97.1	@doofydude	@holleratbianca
	97.2	@_yuridiamorales	@holleratbianca
	97.3	@mellyann_	@holleratbianca
98	98	@umanamd	
	98.1	@ketaminh	@umanamd
	98.2	@thetechdoc	@umanamd
	98.3	@njoshi8	@umanamd
99	99	@sofbasauri	
	99.1	@ecctrainings	@sofbasauri
	99.2	@gflores911	@sofbasauri
	99.3	@naemtadvocacypr	@sofbasauri
100	100	@foamnglow	
	100.1	@foamnglow	@foamnglow
	100.1.1	@mogann_davis	@foamnglow
	100.1.2	@_marissa_lynn_	@foamnglow
101	101	@foam_highlights	
	101.1	@chriscarrollmd	@foam_highlights
	101.2	@ilindelatorremd	@foam_highlights
	101.3	@ilindelatorremd	@foam_highlights
102	102	@tmit2	
	102.1	@kosherdocoz	@tmit2
	102.2	@sandnsurf	@tmit2
	102.2.1	@docnikko	@sandnsurf
103	103	@srrezaie	
	103.1	@medicalabacus	@srrezaie
	103.2	@erbrad	@srrezaie
	103.3	@paramedickiwi	@srrezaie
104	104	@emswami	
	104.1	@jdfried	@emswami
	104.1.1	@tchanmd	@jdfried
	104.2	@tchanmd	@emswami
105	105	@ultrasoundjelly	
	105.1	@ultrasoundrel	@ultrasoundjelly
	105.2	@emhighak	@ultrasoundjelly
	105.2.1	@ultrasoundmd	@emhighak

106	106	@foamnglow	
	106.1	@jaythaprophet	@foamnglow
	106.2	@casey_huett	@foamnglow
	106.3	@livkoz	@foamnglow
107	107	@srrezaie	
	107.1	@smaccrun	@srrezaie
	107.2	@me7sn	@srrezaie
	107.3	@bcm_emig	@srrezaie
108	108	@emeducation	
	108.1	@academicnation	@emeducation
	108.2	@ecctrainings	@emeducation
	108.3	@gflores911	@emeducation
109	109	@ultrasoundrel	
	109.1	@ultrasoundrel	@ultrasoundrel
	109.2	@ultrasoundrel	@ultrasoundrel
	109.3	@ultrasoundrel	@ultrasoundrel
110	110	@clinicalcaserev	
	110.1	@clinicalcaserev	@clinicalcaserev
	110.2	@clinicalcaserev	@clinicalcaserev
	110.3	@clinicalcaserev	@clinicalcaserev
111	111	@ambofoam	
	111.1	@kangaroobeach	@ambofoam
	111.1.1	@drmedmonds	@kangaroobeach
	111.1.1.1	@kangaroobeach	@drmedmonds
112	112	@maitiu78	
	112.1	@vicpain1	@maitiu78
	112.2	@broomedocs	@maitiu78
	112.3	@judkinssimon	@maitiu78
113	113	@clinicalcaserev	
	113.1	@marysandum	@clinicalcaserev
	113.2	@larizamarz25	@clinicalcaserev
	113.3	@alhasilnan	@clinicalcaserev
114	114	@clinicalcaserev	
	114.1	@sophiaperlick	@clinicalcaserev
	114.2	@clinicalcaserev	@clinicalcaserev
	114.2.1	@larizamarz25	@clinicalcaserev
115	115	@chartierlucas	
	115.1	@eusmd	@chartierlucas
	115.2	@humanfact0rz	@chartierlucas
	115.2.1	@eusmd	@humanfact0rz
116	116	@emmanchester	

	116.1	@emmanchester	@emmanchester
	116.2	@spaynesarah	@emmanchester
	116.2.1	@emmanchester	@spaynesarah
117	117	@clinicalcaserev	
	117.1	@clinicalcaserev	@clinicalcaserev
	117.2	@clinicalcaserev	@clinicalcaserev
	117.2.1	@allenwellmd	@clinicalcaserev
118	118	@vpimedic	
	118.1	@embasic	@vpimedic
	118.2	@chanel5	@vpimedic
	118.3	@werecruitdrs	@vpimedic
119	119	@ermentor	
	119.1	@serkane_eroglu	@ermentor
	119.2	@jameslhuffman	@ermentor
	119.3	@ilindelatorremd	@ermentor
120	120	@brent_thoma	
	120.1	@emlitofnote	@brent_thoma
	120.2	@sandnsurf	@brent_thoma
	120.2.1	@brent_thoma	@sandnsurf
121	121	@hp_ems	
	121.1	@serkane_eroglu	@hp_ems
	121.2	@gflores911	@hp_ems
	121.3	@ecctrainings	@hp_ems
122	122	@jake_turner2503	
	122.1	@paramedic_tutor	@jake_turner2503
	122.2	@paramedickiwi	@jake_turner2503
	122.3	@emergmedottawa	@jake_turner2503
123	123	@clinicalcaserev	
	123.1	@dominicanyummd	@clinicalcaserev
	123.2	@sophiaperlick	@clinicalcaserev
	123.3	@clinicalcaserev	@clinicalcaserev
124	124	@orlandopavezmd	@drbastuardo
	124.1	@drbastuardo	@orlandopavezmd
	124.2	@drbastuardo	@orlandopavezmd
	124.2.1	@drbastuardo	@drbastuardo
125	125	@clinicalcaserev	
	125.1	@drakates	@clinicalcaserev
	125.2	@drmariosaa	@clinicalcaserev
	125.3	@dannysafarimd	@clinicalcaserev
126	126	@clinicalcaserev	
	126.1	@dominicanyummd	@clinicalcaserev

	126.2	@clinicalcaserev	@clinicalcaserev
	126.2.1	@dominicanyummd	@clinicalcaserev
127	127	@lwestafer	
	127.1	@_nmay	@lwestafer
	127.1.1	@nvdwaa	@_nmay
	127.2	@nickjohnsonmd	@lwestafer
128	128	@thesgem	
	128.1	@thinkalot	@thesgem
	128.1.1	@thesgem	@thinkalot
	128.2	@rollcagemedic	@thesgem
129	129	@alittlemedic	
	129.1	@ambofoam	@alittlemedic
	129.1.1	@ambofoam	@ambofoam
	129.2	@traumajunkie17	@alittlemedic
130	130	@clinicalcaserev	
	130.1	@drmariosaa	@clinicalcaserev
	130.2	@drdariob	@clinicalcaserev
	130.3	@drsamwhite	@clinicalcaserev
131	131	@ketaminh	
	131.1	@mdaware	@ketaminh
	131.1.1	@ebmgonewild	@mdaware
	131.1.2	@broomedocs	@mdaware
132	132	@allanmix	
	132.1	@ecctrainings	@allanmix
	132.2	@gflores911	@allanmix
	132.2.1	@thinkalot	@gflores911
133	133	@clinicalcaserev	
	133.1	@stefanoamir	@clinicalcaserev
	133.2	@dominicanyummd	@clinicalcaserev
	133.3	@clinicalcaserev	@clinicalcaserev
134	134	@wikem_org	
	134.1	@embasic	@wikem_org
	134.2	@jimiyates	@wikem_org
	134.3	@xrawanx	@wikem_org
135	135	@chriscarrollmd	
	135.1	@thomasevans435	@chriscarrollmd
	135.2	@texaskiddoc	@chriscarrollmd
	135.3	@drjengunter	@chriscarrollmd
136	136	@fluffmed	
	136.1	@andywebster	@fluffmed
	136.2	@mal_197	@fluffmed

	136.3	@ivline	@fluffmed
137	137	@clinicalcaserev	
	137.1	@clinicalcaserev	@clinicalcaserev
	137.2	@clinicalcaserev	@clinicalcaserev
	137.3	@clinicalcaserev	@clinicalcaserev
138	138	@ebmgonewild	
	138.1	@kangaroobeach	@ebmgonewild
	138.2	@umanamd	@ebmgonewild
	138.3	@ermentor	@ebmgonewild
139	139	@jojohaber	
	139.1	@srrezaie	@jojohaber
	139.2	@srrezaie	@jojohaber
	139.3	@srrezaie	@jojohaber
140	140	@srrezaie	
	140.1	@acmt	@srrezaie
	140.2	@usmlethree	@srrezaie
	140.3	@ditchdocrn14	@srrezaie
141	141	@smithecgblog	
	141.1	@medquestioning	@smithecgblog
	141.2	@emmanchester	@smithecgblog
	141.2.1	@ponderingpaeds	@emmanchester
142	142	@ojedathies	
	142.1	@ojedathies	@ojedathies
	142.2	@ojedathies	@ojedathies
	142.3	@ojedathies	@ojedathies
143	143	@erspublications	
	143.1	@tobyhillman	@erspublications
	143.2	@skimmingstones1	@erspublications
	143.3	@thechestreg	@erspublications
144	144	@drwaisun	
	144.1	@cg_dr	@drwaisun
	144.2	@ndyg83	@drwaisun
	144.3	@mkchan_rcpsc	@drwaisun
145	145	@srrezaie	
	145.1	@mesharialhamed1	@srrezaie
	145.2	@jacobykatherine	@srrezaie
	145.3	@kubed	@srrezaie
146	146	@precordialthump	
	146.1	@cjpoynter	@precordialthump
	146.2	@mlalanda	@precordialthump
	146.2.1	@precordialthump	@mlalanda

147	147	@emcases	
	147.1	@precordialthump	@emcases
	147.1.1	@kirstychallen	@precordialthump
	147.2	@ettube	@emcases
148	148	@srrezaie	
	148.1	@ilindelatorremd	@srrezaie
	148.2	@roxicet_rn	@srrezaie
	148.3	@ilindelatorremd	@srrezaie
149	149	@umanamd	
	149.1	@emilyannmaier	@umanamd
	149.2	@jnichol4077	@umanamd
	149.3	@petrosoniak	@umanamd
150	150	@em_resus	
	150.1	@twhitingem	@em_resus
	150.2	@damian_roland	@em_resus
	150.3	@edtakedown	@em_resus
