# Supplementary material for: Preliminary Evidence for the Emergence of a Health Care Online Community of Practice: Using a Netnographic Framework for Twitter Hashtag Analytics
Source: J Med Internet Res. 2017 Jul 14;19(7):e252. doi: 10.2196/jmir.7072 (PMC5533942; doi:10.2196/jmir.7072)
Supplement: Multimedia Appendix 4 [file jmir_v19i7e252_app4.pdf]

Top 100 links shared by the #FOAMed community

No. of shares	URL
941	http://rebelem.com/rebel-reviews/
802	http://thesgem.com/
680	http://wellingtonicu.com/Data/Antibiotic%20Overview.pdf
550	http://www.rcemfoamed.co.uk/
285	http://intensivecarenetwork.com/media/
277	http://www.heftemcast.co.uk/
259	https://mobile.twitter.com/srrezaie/status/393007422089859072/photo/1
227	http://www.ccforum.com/content/19/1/251
222	http://EMin5.com/
220	http://emblog.mayo.edu/
208	http://www.clinicalcasereview.org/mnemonics-.html[a]
196	http://www.emdocs.net/
196	http://rebelem.com/a-new-pulseless-electrical-activity-algorithm/
195	https://itunes.apple.com/gb/podcast/cem-foamed-network/id898228684?mt=2
193	http://rebelem.com/rebel-reviews
191	http://www.atacc.co.uk/e-learning/
189	http://www.nejm.org/doi/full/10.1056/NEJMoa1411099
182	https://mobile.twitter.com/EM_ResUS/status/619883691230298112/photo/1
178	http://www.learntheheart.com/ecg-review/ecg-interpretation-tutorial/introduction-to-the-ecg/
169	https://itunes.apple.com/us/podcast/foamcast/id879281888?mt=2
168	http://rebelem.com/preoxygenation-apneic-oxygenation/
165	http://www.emdocs.net/furosemide-treatment-acute-pulmonary-edema/
164	https://mobile.twitter.com/EM_ResUS/status/595986794786742272/photo/1
161	http://www.aliem.com/em-im-residency-on-twitter/
160	http://www.nejm.org/doi/full/10.1056/NEJMoa1405796
152	https://mobile.twitter.com/EM_ResUS/status/556914367800025088/photo/1
150	http://www.ncbi.nlm.nih.gov/pmc/articles/PMC4127295/
150	https://www.meded101.com/case-study-zocor-and-diltiazem-interaction/
148	http://PEMgeek.com/
148	http://rebelem.com/ecg-changes-hyperkalemia/
146	http://boringem.org/2015/02/05/nice-threads-guide-suture-choice-ed/
142	http://radiopaedia.org/cases/april-fools-2015-ectopia-cordis-interna-tin-man-syndrome
142	https://itunes.apple.com/us/book/introduction-to-bedside-ultrasound/id554196012?mt=11
137	http://www.learntheheart.com/blogs/congestive-heart-failure-volume-fluid-status/
134	http://mediwikis.com/wiki/index.php/Category:Neurology?
132	http://rolobotrambles.com/2014/10/12/the-path-to-developing-f-o-a-m-free-open-access-meducation-foamed/
131	https://itunes.apple.com/gb/podcast/heft-emcast/id751375884?mt=2
130	http://www.meded101.com/
130	http://www.pemcincinnati.com/blog/
130	http://rebelem.com/heart-score-new-ed-chest-pain-risk-stratification-score/
125	http://rebelem.com/chest-pain-value-good-history/
123	http://theteachingcourse.com/
123	http://www.tamingthesru.com/blog/grand-rounds/im-leaving
123	http://chronicle.com/search/?

	search_siteId=5&contextId=&action=rem&searchQueryString=robert+sternberg
122	http://www.learntheheart.com/cardiology-review/clinical-trials/atrial-fibrillation/
122	http://www.acep.org/Physician-Resources/Policies/Policy-Statements/EMS-Management-of-Patients-with-Potential-Spinal-Injury/#FOAMed[a]
120	http://www.emdocs.net/myths-dka-management/
120	http://www.scancrit.com/2015/07/12/archaic-trauma-life-support/?
120	http://www.scancrit.com/2015/02/12/cervical-collars-slashed-guidelines/
119	http://stemlynsblog.org/button-batteries/
116	http://www.ncbi.nlm.nih.gov/pmc/articles/PMC3613190/
111	http://www.paic.com.au/index.php/workshops
110	https://www.youtube.com/watch?v=eSEP2T-xz8g&feature=youtu.be
110	http://knowmedge.com/blog/oncology-pearls-abim-internal-medicine-shelf-exams/
110	http://onlinelibrary.wiley.com/doi/10.1111/1742-6723.12361/abstract
109	http://www.learntheheart.com/cardiology-review/coronary-artery-disease-stemi/
108	http://iteachem.net/2013/06/ten-tips-for-foam-beginners/
108	https://itunes.apple.com/us/book/practical-ultrasound-series/id919137205?mt=11
107	http://www.ccforum.com/search/results?drpField1=[AT]&txtSearch1=Review
106	http://injectableorange.com/2015/07/respiratory-review-principles-of-mechanical-ventilation/
106	http://rebelem.com/importance-reciprocal-changes-avl/
105	http://www.learntheheart.com/atrial-fibrillation-guidelines/
104	http://www.ultrasoundoftheweek.com/
103	http://paper.li/ICU_BUFALINI/1418809455
103	http://www.emdocs.net/the-approach-to-the-poisoned-patient/
102	http://www.wessexics.com/The_Bottom_Line/Review/index.php?id=3665078336903245716
101	http://www.mededmasters.com/case-of-the-week.html[a]
101	https://itunes.apple.com/us/book/introduction-to-bedside-ultrasound/id647356692?mt=11
101	http://www.wessexics.com/The_Bottom_Line/
100	http://livestream.com/iTeachEM/events/2539760
100	https://docs.google.com/forms/d/1A5bcPoW3It79UTWMvZ1Emx2xmONapHSHI0kwlc-heGM/viewform?c=0&w=1&usp=send_form
99	http://rebelem.com/benefit-initial-insulin-bolus-diabetic-ketoacidosis/
99	http://www.ccforum.com/content/19/1/175
99	http://hqmeded-ecg.blogspot.com/2015/04/pulseless-ventricular-tachycardia-why.html
99	http://www.nejm.org/doi/full/10.1056/NEJMe1503623
99	http://knowmedge.com/blog/5-high-yield-cardiology-pearls-help-pass-internal-medicine-boards-2/
99	http://rebelem.com/time-abandon-epinephrine-hospital-cardiac-arrest/
99	https://vine.co/v/e1WBD9dL2Vz
98	https://mobile.twitter.com/EM_ResUS/status/627485559439654912/photo/1
98	http://www.learntheheart.com/ecg-dance/
98	http://foamcast.org/2015/05/04/episode-28-neuroleptic-malignant-syndrome-serotonin-syndrome-malignant-hyperthermia/
97	http://www.lasvegasemr.com/foam-blog
95	https://www.youtube.com/channel/UCtaRF58UDVthvH36YYCttng
94	http://www.guidelinesforme.com/
93	http://www.aliem.com/intraosseous-rapid-sequence-intubation/
92	http://www.ncbi.nlm.nih.gov/pubmed/?term=25536868
92	http://www.aliem.com/upper-gastrointestinal-bleeding-treatment/

91	http://rebelem.com/patients-strep-throat-need-treated-antibiotics/
91	http://lifeinthefastlane.com/5-lessons-learned/
91	http://emergencymedicineireland.com/2012/02/anatomy-for-emergency-medicine-7-cricothyroidotomy/
90	https://twitter.com/Brodalumab/status/465044302867873792/photo/1[a]
90	http://www.ncbi.nlm.nih.gov/pubmed/24556776
89	http://www.nejm.org/doi/full/10.1056/NEJMoa1404380
89	http://thesgem.com/2014/04/thrombolysis-for-acute-stroke/
89	http://www.cmajopen.ca/content/3/2/E166.full
89	http://rebelem.com/
88	https://twitter.com/srrezaie/status/393007422089859072/photo/1
88	http://www.cemfoamed.co.uk/
87	http://www.ccforum.com/content/19/1/186
87	http://www.pulmcrit.org/2015/03/management-of-severe-hyperkalemia-in.html?m=1[a]

[a]Link is no longer active as of December 2015.
