# Supplementary material for: Preliminary Evidence for the Emergence of a Health Care Online Community of Practice: Using a Netnographic Framework for Twitter Hashtag Analytics
Source: J Med Internet Res. 2017 Jul 14;19(7):e252. doi: 10.2196/jmir.7072 (PMC5533942; doi:10.2196/jmir.7072)
Supplement: Multimedia Appendix 5 [file jmir_v19i7e252_app5.pdf]

Netnographic evaluation of digital products from #FOAMed conversations.

URL	Reviewer engagement data	Inferred meaning
http://rebelem.com/a-new-pulseless-electrical-activity-algorithm/	11 comments<br>5 trackbacks[a] from other FOAM[b] blogs	Discussion thread between commenters and authors demonstrated critique and reflection on a new paradigm presented in the post.<br>Comments inferred exploration of testing the ideas in individual practice contexts and discussing strengths and weaknesses.<br>Trackbacks showed the nodal relationship of blogs and individual members of the network.
http://rebelem.com/preoxygenation-apneic-oxygenation/	8 comments<br>3 trackbacks from other FOAM blogs	General comments of thanks and seeking permission to share further with colleagues to influence practice change.<br>Trackbacks showed the nodal relationship of blogs and the individual members of the network.
http://www.emdocs.net/furosemide-treatment-acute-pulmonary-edema/	24 comments<br>8 trackbacks from other FOAM blogs	Robust discussion and assertions of competing evidence or literature; a clear example of a socially constructed dialogue around a blog that presented evidence that challenged some commenters' current practices, while validating others'.<br>Built upon a blog initially focusing on incorporating evidence from other therapies and clinical experiences.<br><br>Trackbacks showed the nodal relationship of blogs and the individual members of the network.
https://www.meded101.com/case-study-zocor-and-diltiazem-interaction/	5 comments	Comments inferred a social process of making sense of the assertions of the blog in the face of anecdotal clinical experience.
http://rebelem.com/ecg-changes-hyperkalemia/	10 comments<br>1 trackback from another FOAM blog	Comments explored and refined the concepts from the blog and commenters appeared to be trying to draw links to their clinical contexts.<br>There was a strong evident critique and reframing of the blog comments by 2 commenters, evidencing social construction within a tacit experience framework.<br>Trackbacks showed the nodal relationship of blogs and the individual members of the network.
http://boringem.org/2015/02/05/nice-threads-guide-suture-choice-ed/	3 comments	A correction by a commenter led to immediate revision of the blog, inferring a strong mechanism of social moderation and change.
http://rolobotrambles.com/2014/10/12/the-path-to-developing-f-o-a-m-free-open-access-meducation-foamed/	1 comment<br>3 trackbacks from other FOAM blogs	Affirmation of blog, with some social refinement of concepts based on the philosophy and reading of the commenter.<br>Trackbacks showed the nodal relationship of blogs and the individual members of the network.
http://rebelem.com/hear	6 trackbacks from other	Trackbacks showed the nodal relationship of blogs and

t-score-new-ed-chest-pain-risk-stratification-score/	FOAM blogs	the individual members of the network.
http://rebelem.com/chest-pain-value-good-history/	2 comments<br>2 trackbacks from other FOAM blogs	A question-and-answer exchange to seek placement of the knowledge from the blog into a tacit clinical context and frame within a clinical decision-making framework.<br>Trackbacks showed the nodal relationship of blogs and the individual members of the network.
http://www.emdocs.net/myths-dka-management/	7 comments<br>4 trackbacks from other FOAM blogs	Further questions and commenters seeking how to translate the content of the blog into clinical practice.<br>Trackbacks showed the nodal relationship of blogs and the individual members of the network.
http://www.scancrit.com/2015/07/12/archaic-trauma-life-support/?	2 comments<br>1 trackback link from another FOAM blog	Affirmation and solidarity over the perceived need to challenge established institution.<br>Trackbacks showed the nodal relationship of blogs and the individual members of the network.
http://www.scancrit.com/2015/02/12/cervical-collars-slashed-guidelines/	13 comments<br>3 trackbacks from other FOAM blogs	A strongly worded discussion on a topic that clearly created cognitive dissonance for some of the commenters.<br>The comments reflected the early clinical discussions around the challenges to long-term established and belief-driven practice.<br>A very strong example of social moderation of potentially practice-changing knowledge.<br>Trackbacks showed the nodal relationship of blogs and the individual members of the network.
http://stemlynsblog.org/button-batteries/	6 comments<br>2 trackbacks from other FOAM blogs	Comments reflected affirmation and the socially constructed beginnings of an awareness-raising movement.<br>Trackbacks showed the nodal relationship of blogs and the individual members of the network.
https://www.youtube.com/watch?v=eSEP2T-xz8g&feature=youtu.be	Sketchy evidence-based medicine “How to read a paper” video<br>1 comment	Affirmation and outreach for collaboration.
http://iteachem.net/2013/06/ten-tips-for-foam-beginners/	1 comment<br>1 trackback link from another FOAM blog	Affirmation of blog content and description of how the user will promulgate it to his practice network.<br>Trackbacks showed the nodal relationship of blogs and the individual members of the network.
http://rebelem.com/importance-reciprocal-changes-avl/	2 comments	Constructive feedback from an expert in the clinical topic, providing tacit knowledge context to the blog.<br>Author accepting critique and thanking critics and affirming the comments.
http://www.emdocs.net/the-approach-to-the-poisoned-patient/	1 comment	Affirmation for blog content as being the most relevant and well-structured approach to the poisoned patient the commenter had seen in 6 years of emergency medicine practice.
http://www.wessexics.com/The_Bottom_Line/	6 comments	Discussion thread elaborating on the clinical translations, with clear comment as to how the

Review/index.php?id=3665078336903245716		findings of the research reviewed will translate to change clinical practice.
http://rebelem.com/benefit-initial-insulin-bolus-diabetic-ketoacidosis/	10 comments	Affirmation of blog post quality; 1 commenter said that the post validated his clinical practice in diabetic ketoacidosis management.<br>Another commenter inferred that the content would lead to a change of diabetic ketoacidosis management in his “order set.”<br>Also further skeptical discussion of the primary literature reviewed in the post.
http://hqmeded-ecg.blogspot.com/2015/04/pulseless-ventricular-tachycardia-why.html	2 comments	Affirmation, translation, and reflection from commenters’ own clinical practice contexts, with feedback on how to overcome one of the clinical issues from the case presented.
http://rebelem.com/time-abandon-epinephrine-hospital-cardiac-arrest/	4 comments<br>3 trackbacks from other FOAM blogs	Discussion thread and links to further evidence and forthcoming trials on the topic.<br>Showed tangential expansion through community input.<br>Trackbacks showed the nodal relationship of blogs and the individual members of the network.
http://foamcast.org/2015/05/04/episode-28-neuroleptic-malignant-syndrome-serotonin-syndrome-malignant-hyperthermia/	2 comments<br>2 trackbacks from other FOAM blogs	Affirmation of quality and content.<br>Trackbacks showed the nodal relationship of blogs and the individual members of the network.
http://www.aliem.com/intraosseous-rapid-sequence-intubation/	7 comments	Discussion and links to further literature.<br>Debate from the perspective of practice area.<br>Clarification and elaboration from the author of the primary literature reviewed in the blog post.
http://www.aliem.com/upper-gastrointestinal-bleeding-treatment/	9 comments	Combination of affirmation and further links and offerings of evidence to build upon the blog post.
http://rebelem.com/patients-strep-throat-need-treated-antibiotics/	12 comments	Discussion with author including moderation, elaboration, and extrapolation of discussion.<br>Questions from commenters help to refine and contextualize the practices discussed in the blog and moderated assertions dependent on patient population.
http://lifeinthefastlane.com/5-lessons-learned/	28 comments<br>1 trackback from another FOAM blog	A reflective blog piece.<br>Comments offered social support, affirmation, and reflection upon commenters’ own experiences.<br>Trackbacks showed the nodal relationship of blogs and the individual members of the network.
http://emergencymedicineireland.com/2012/02/anatomy-for-emergency-medicine-7-cricothyroidotomy/	1 comment<br>6 trackbacks from other FOAM blogs	A comment of thanks.<br>Trackbacks showed the nodal relationship of blogs and the individual members of the network.

[a]Trackbacks are defined as links directing to other pages.

[b]FOAM: free open access medical education.
